# Intestinal Permeability in Children with Celiac Disease after the Administration of Oligofructose-Enriched Inulin into a Gluten-Free Diet—Results of a Randomized, Placebo-Controlled, Pilot Trial

**DOI:** 10.3390/nu12061736

**Published:** 2020-06-10

**Authors:** Natalia Drabińska, Urszula Krupa-Kozak, Elżbieta Jarocka-Cyrta

**Affiliations:** 1 Department of Chemistry and Biodynamics of Food, Institute of Animal Reproduction and Food Research of Polish Academy of Sciences, 10-748 Olsztyn, Poland; n.drabinska@pan.olsztyn.pl; 2 Department of Pediatrics, Gastroenterology, and Nutrition, Collegium Medicum, University of Warmia & Mazury, 10-719 Olsztyn, Poland

**Keywords:** intestinal permeability, leaky gut, gut barrier, celiac disease, prebiotic, gluten-free diet, randomized controlled trial, gluten immunogenic peptides, sugar absorption test

## Abstract

Abnormalities in the intestinal barrier are a possible cause of celiac disease (CD) development. In animal studies, the positive effect of prebiotics on the improvement of gut barrier parameters has been observed, but the results of human studies to date remain inconsistent. Therefore, this study aimed to evaluate the effect of twelve-week supplementation of a gluten-free diet (GFD) with prebiotic oligofructose-enriched inulin (10 g per day) on the intestinal permeability in children with CD treated with a GFD. A pilot, randomized, placebo-controlled nutritional intervention was conducted in 34 children with CD, being on a strict GFD. Sugar absorption test (SAT) and the concentrations of intestinal permeability markers, such as zonulin, intestinal fatty acid-binding protein, claudin-3, calprotectin, and glucagon-like peptide-2, were measured. We found that the supplementation with prebiotic did not have a substantial effect on barrier integrity. Prebiotic intake increased excretion of mannitol, which may suggest an increase in the epithelial surface. Most children in our study seem to have normal values for intestinal permeability tests before the intervention. For individuals with elevated values, improvement in calprotectin and SAT was observed after the prebiotic intake. This preliminary study suggests that prebiotics may have an impact on the intestinal barrier, but it requires confirmation in studies with more subjects with ongoing leaky gut.

## 1. Introduction

The intestinal barrier is a complex structure which separates the external environment from the internal milieu of the human body. This physical, selective barrier allows for the passage of essential ions, water, nutrients, and immune mediators, and, at the same time, strongly restricts the transport of potentially harmful microbes and antigens. The intestinal barrier plays a pivotal role in human body homeostasis, being responsible for digestion, absorption, and motility, as well as endocrine and immunological functions, of the intestinal tract [1]. The gut barrier consists of three main compartments: gut-associated lymphoid tissue containing various immune cells; the epithelium; and the intestinal microbiota [2]. The epithelium of small intestine is a monolayer containing different specialized cells, such as enterocytes, goblet cells, Paneth cells, endocrine cells, and intestinal stem cells, which are sealed tightly together by intercellular junctions [2]. These intercellular junctions are composed of several proteins, including occludin, claudins, and zonula occludens. In physiological conditions, intestinal epithelial cells are in constant turnover, renewing and replacing all the cells in a few days [3], and the functioning of the barrier restores itself even after short periods of ischemia [4].

Recently, it was suggested that chronic disruptions in the functioning of the intestinal barrier can participate in the pathogenesis of several disorders, including autoimmune diseases [1,5,6]. In the altered barrier, non-competent intercellular junctions allow antigens derived from the food or intestinal microbiota to enter the internal milieu which challenges the immune system, resulting in the upregulated immune response. It is considered that the dysfunction of the intestinal barrier is one of the factors involved in the pathogenesis of the celiac disease (CD) [2]. CD is a chronic, systemic, autoimmune disease manifested by small intestine enteropathy and a combination of various intestinal and extraintestinal symptoms. The key, well-defined players in CD pathogenesis are a genetic factor (human leukocyte antigen (HLA) DQ 2 and HLA DQ8), the environmental trigger (gluten prolamines), and autoantigen involvement (tissue transglutaminase 2 (tTG)). However, recently, new factors were suggested for CD developments, including gut microbiota dysbiosis and early disruption of the gut barrier [2]. A recent study showed the altered expression of genes involved in the functioning of epithelial cells in CD, confirming the importance of maintenance of the intestinal barrier in CD [5].

The insight into the gut barrier integrity is imperative for clinical practice and understanding of the development and course of several diseases. Several methods have been implemented to analyze the intestinal permeability. One of the commonly used functional assessment of barrier function is sugar absorption test (SAT), which is based on the assumption that the orally ingested large molecular sugar cannot cross the barrier unless its function is compromised [7]. The most commonly used sugars for the SAT are lactulose (L) and smaller mannitol (M) [8,9,10,11]. The amount of M, as a readily-absorbed monomer, informs about the trans-cellular uptake and an intact epithelial surface area, while the percent of L recovery reflects dysfunction of the intestinal barrier due to compromised intercellular junctions or reflects the areas of mucosal epithelial breach [12]. Besides the functional tests, several non-invasive biomarkers associated with epithelial cell damage, such as intestinal fatty acid-binding protein (iFABP), citrulline, zonulin, and claudins, as well as markers related to inflammation, such as calprotectin and alpha-1-antitrypsin, were proposed [13,14,15]. To date, no universal marker can give a definitive answer on the permeability of the gut; therefore, the combination of the methods can provide better insight into the gut integrity.

Several studies showed that selected strains of bacteria can positively influence the gut barrier [16,17]. Moreover, it is suggested that food components can strengthen the integrity of the gut barrier, mostly by affecting the gut microbiota and restoring the microbial homeostasis [18,19,20]. Therefore, prebiotics, the substrates that are selectively utilized by host microorganisms conferring a health benefit [21], were proposed as a promising intervention for gut barrier improvement [22,23]. The positive effects of prebiotics and prebiotic-derived metabolites on the gut barrier functions have been repeatedly confirmed by in vitro and in vivo studies [24,25,26]. However, there is no agreement in the influence of prebiotics in clinical trials. On the one hand, there was no influence of prebiotics on the intestinal permeability reported in several groups of patients [27,28,29], while, in other studies, the positive effect of prebiotics was suggested [30]. However, to date, there are no studies on the effect of prebiotics on the intestinal barrier in CD. In our previous study, we observed the changes in the concentrations of amino acids involved in the gut barrier integrity, glutamic acid, and its precursor glutamine [31], which inspired us to further evaluate the gut barrier functions. Therefore, the aim of this study was to evaluate the effect of twelve-week supplementation of a gluten-free diet (GFD) with prebiotic oligofructose-enriched inulin on the intestinal permeability in children and adolescents with CD.

## 2. Materials and Methods

### 2.1. Study Protocol

Thirty-four children with diagnosed CD, following GFD regime for at least six months, were recruited among the patients of the Gastrointestinal Clinic in the Children’s Hospital in Olsztyn (Poland) to participate in a randomized, placebo-controlled, single-center clinical trial with the nutritional intervention registered in the U.S. National Library of Medicine (ID: NCT03064997; http://www.clinicaltrials.gov) [32]. Participants were randomly assigned to a group receiving 10 g of oligofructose-enriched inulin (Synergy 1; Orafti^®^, Beneo, Belgium) per day or a group receiving placebo (maltodextrin) for twelve weeks. The study was performed from January to June 2016. The full details of the patients characteristic, study protocol, inclusion/exclusion criteria, and a Consolidated Standards of Reporting Trials (CONSORT) chart are described elsewhere [33]. Participants and their caregivers, clinicians, and most of the investigators (except one person providing supplements) were blinded. The results describing blood morphology, intestinal microbiota, amino acid metabolism, the status of fat-soluble vitamins, and bone metabolism were previously published as separate manuscripts [31,34,35,36,37].

Parents or caregivers of participants were fully informed about the study and signed the written informed consent on the first check-up visit. The study protocol has been approved by the Bioethics Committee of the Faculty of Medicine of the University of Warmia and Mazury in Olsztyn, Poland (decision No. 23/2015). All procedures involving human participants were performed with the ethical principles of the 1964 Declaration of Helsinki and its later amendments.

### 2.2. Sugar Absorption Test (SAT)

Subjects fasted overnight, and, after emptying their bladder, they subsequently ingested a sugar mixture containing 10 g of L and 2 g of M mixed into approximately 250 mL of water and then were instructed to collect urine for 5 h. Urine samples were transferred to the urine containers with 100 μL of 1% chlorhexidine diacetate (Pharma Cosmetic, Kraków, Poland) to prevent bacterial growth. The volume of the urine collections was recorded, and aliquots were stored at −80 °C until further analysis.

The recoveries of L and M were analyzed using a modified method of Houben et al. [38]. Briefly, 200 μL of urine was evaporated to dryness at 64 °C. Next, 50 μL of MOX reagent (methoxyamine hydrochloride in pyridine) and 70 μL of pyridine were added, mixed, and heated at 75 °C. After 30 min, the sample was cooled for 10 min in ice bath, and 100 μL N,O-Bis(trimethylsilyl)trifluoroacetamide (BSTFA) +1% trimethylsilyl chloride (TMCS) was added. The mixture was vortexed for 20 s and heated at 75 °C for 35 min. The sample was evaporated to dryness at 64 °C. The residue was dissolved in 250 μL of hexane, and 2 μL was injected into the injection port set at 280 °C.

Derivatized sugars were analyzed using an HP 5890 gas chromatograph coupled with HP 5972 mass selective detector (Agilent Technologies, Santa Clara, CA, USA). Compounds were separated using HP-5MS capillary column, 30 m × 0.25 mm × 0.25 μm (Agilent Technologies, Santa Clara, CA, USA). The oven temperature program was as follows: 100 °C held for 3 min, an increase to 210 °C at a rate of 30 °C/min, then increase to 290 °C at a rate of 15 °C/min and again increase to 310 °C at a rate of 30 °C/min, then held for 5 min, with a total run time of 17 min. The carrier gas was helium at a constant flow of 1.5 mL/min. After a solvent delay of 6 min, mass spectra were obtained by electron ionization (EI) in the range of 40–700 m/z. Transfer line, ion source, and quadrupole temperatures were 280 °C, 230 °C, and 180 °C, respectively. Total ion chromatograms were analyzed with the MSD ChemStation E.02.02.1431 software (Agilent Technologies, Santa Clara, CA, USA). Quantification of L and M was done by external standard calibration.

### 2.3. Biochemical Assays

Biological material (blood plasma and serum, stool) was collected before and after the intervention, as described earlier [33]. The concentration of GLP-2 in plasma was analyzed using commercial Human GLP-2 (Glucagon-Like Peptide 2) ELISA Kit (Elabscience, Bethesda, MD, USA) with a detection range of 0.16–10 ng/mL, the sensitivity of 0.10 ng/mL, and coefficient of variation < 6%. The analysis of plasma zonulin was performed using commercial Human Zonulin ELISA Kit (Elabscience, Bethesda, MD, USA) with a detection range of 0.78–50 ng/mL, the sensitivity of 0.47 ng/mL, and coefficient of variation < 6%. The analysis of iFABP in plasma was performed using commercial Human IFABP/FABP2 (Intestinal Fatty Acid Binding Protein) ELISA Kit (Elabscience, Bethesda, MD, USA) with a detection range of 0.16–10 ng/mL, the sensitivity of 0.17 ng/mL, and coefficient of variation < 7%. The level of claudin-3 was measured in serum using Human ELISA Kit for Claudin 3 (CLDN3) (Cloud-Clone Corp., Houston, TX, USA), with a detection range of 0.312–20ng/mL, the limit of detection of 0.112 ng/mL, and precision parameters below 12%, according to the manufacturer. Calprotectin concentration in stool samples was analyzed using IDK^®^ Calprotectin ELISA Kit (Stool, 1h) CE IVD (Immundiagnostik, Bensheim, Germany) with a linear range of 13–840 ng/mL, the limit of detection of 1.023 ng/mL, and precision parameters below 9%.

### 2.4. Gluten Immunogenic Peptides (GIP)

An analysis of GIP in stool samples was performed by ELISA method using the iVYLISA GIP-S kit (Biomedal DIAGNOSTICS, Sevilla, Spain). Range of analysis for this kit was 0.156–5 µg GIP/g of stool sample with 0.156 being limit of quantification. Manufacturer declared 100% sensitivity of the kit.

### 2.5. Statistical Analysis

Initially, 34 children were studied; however, after the three-month intervention, only 30 children were still included. Of these 30 children, 15 were from the Synergy 1 group and 15 from the placebo group. Children were excluded because of antibiotic use during the intervention (n = 2) and non-compliance (compliance rate lower than 80%) with the recommended daily study product consumption level (n = 2). Moreover, because of the lack of urine volume data in SAT assay provided by caregivers, data from only 24 children (13 from Synergy 1 and 11 from the placebo group) were used in the final analyses.

All the analyses were performed in duplicates. The normal distribution of data was performed using the Kolmogorov-Smirnov test. Data following normal distribution are presented as mean ± standard deviation, while data which do not follow normal distribution are presented as median (P25–P75). Differences in characteristics between groups were tested with the parametric Student’s t-test or the non-parametric Mann–Whitney U test. Differences within groups before and after intervention were determined with the Student’s t-test for paired samples or the Wilcoxon test, as appropriate. Results were considered statistically significant at 5% critical level (*p* < 0.05). Correlations between the analyzed parameters were assessed using the Pearson correlation coefficient test. All statistical analyses were carried out using IBM SPSS statistics version 26.

## 3. Results and Discussion

### 3.1. GIP

The detection of GIP in stool samples can inform about the adherence to the GFD [39]. In our study, before the intervention, in 2 participants (one person from placebo and one from Synergy 1 group), the GIP values exceeded the upper limit of quantification (5 µg GIP/g of feces), suggesting the intake of gluten prolamines. After the intervention lasting twelve weeks, the number of subjects with the elevated GIP increased to 6, among which there were 3 children from the placebo and 3 from Synergy 1 group. Our attention was caught by one participant from the placebo group, who had elevated GIP value in both study intervals. However, the level of anti-tissue transglutaminase antibodies (tTG) in this subject was within the reference range in both study intervals and decreased from 7.15 to 4.83 U/L after a 12-week intervention (data regarding tTG values were presented elsewhere [31]). Therefore, it was not possible to accurately conclude if this person was breaking the GFD regime constantly or accidentally. In the remaining participants, the elevated GIP values could be explained, rather, by an incidental consumption of gluten because their tTG values after the intervention were lower than before and did not exceed the reference value for tTG. Only for one participant with elevated GIP, the tTG value increased from 2.46 to 17.1 U/L, which might indicate prolonged exposure to gluten and failure to follow a GFD.

The recent study showed that adherence to the GFD decreases with time, especially in children older than seven years, since the control of the diet by parents decrease [39]. In our study, there was no tendency related to age. Within six children with higher GIP value after the intervention, one was five years old, and the children below seven years were in minority in our study (five children). The previous study showed that there is no strong correlation between serological tests (tTG and deamidated gliadin peptide antibodies, DGP) and the presence of GIP in stool [39]. The level of tTG had prolonged response to gluten intake, both for elevation and decreasing. Even though the GIP test seems to be much more sensitive as compared to serological tests because the response is immediate, not prolonged in time, the one limitation is that it informs only about the intake of gluten up to 72 h after the incidence [40]. Therefore, GIP would have to be analyzed very frequently to confirm if gluten was ingested voluntarily or accidentally and in combination to serological tests informing about long-term diet routine.

### 3.2. Sugar Absorption Test

**Figure 1:**
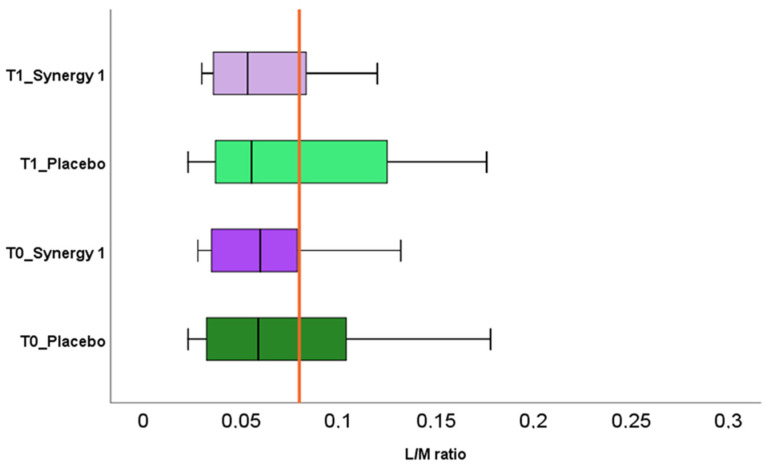
Urinary lactulose (L) to mannitol (M) ratio in Synergy 1 and placebo group, before (T0) and after the intervention (T1). The red line indicates the reference value (0.08).

Most of the studies consider the L/M value of 0.03 as a cut-point for intestinal permeability [9,10,41]. Other studies use a value of 0.09 as a reference, observed in healthy individuals [11,42]. Therefore, because of these discrepancies, in our study, we adopted a reference value of L/M ratio > 0.08 as an indication of intestinal permeability, following the literature data referring to children with CD [8]. The results of L/M before and after the intervention are presented in Figure 1. No significant difference was observed between the experimental groups at enrollment (T0) and after the intervention (T1), nor within the group (Figure 1). Only small, non-significant decreases were observed in medial values of L/M in both, Synergy 1 (0.060 vs. 0.054) and placebo (0.063 vs. 0.056) groups after the intervention. It suggests that both twelve-week supplementation nor the GFD itself had no relevant impact on the intestinal permeability. What is important, in our study, is that the medial values of L/M in both experimental groups were within the normal range reported by Gatti et al. [8]. Based on these results, we can assume that increased permeability of the intestinal barrier was not observed in our study. Our results are in agreement with the results reported by Ho et al. [43], who reported a lack of effect of prebiotic supplementation on intestinal permeability assessed by SAT in children with type 1 diabetes. Inconsistent results were obtained in the study with obese individuals supplemented with arabinoxylans [44]. The authors reported no changes in the intestinal permeability assessed by sugar absorption test (SAT) and the expression of tight junctions proteins. However, the increase in the gene transcription of occludins and claudins, involved in tight junctions functioning, was observed in the group consuming prebiotic [44].

However, the intestinal permeability results of individual subjects participating in this pilot study were interesting. Before the intervention, the elevated value of L/M was detected in six children (four from the placebo group and two from the Synergy 1 group). It is worth emphasizing that all children participating in our study were treated with a GFD since at least six months; therefore, the healing of intestine was expected. After the intervention, the L/M values decreased in two participants from the Synergy 1 group, reaching the normal range. Nevertheless, the elevated L/M values were observed in the other three participants from Synergy 1 group, who had the initial L/M value within a reference range. In the placebo group, after the twelve-week intervention, the decrease in L/M ratio was observed in one child, while, in three other children, no difference was observed. In our study, the effect of prebiotic on intestinal permeability was not observed. This situation may result from the fact that all participants have been treated with a GFD for a relatively long time. Based on the SAT, in most of the children, we did not observe the intestinal leakage that suggested partial or total healing of the intestinal epithelium. Therefore, the results reported by Russo et al. [30] are interesting, as the authors described the improvement of the intestinal permeability after the consumption of inulin-enriched pasta in the healthy subject, who had no history of intestinal diseases nor abnormalities and, in particular, no leaky gut.

The principle of the sugar absorption test is that M is absorbed across cell membranes, while L, as a much bigger molecule, is absorbed through cell junctions. The hypothesis of the permeation of sugars has been provided by Fihn et al. [45]. The authors explained that on top of each villus, there are small but abundant channels allowing for the transfer of M, while in crypts, there are much bigger channels in low abundance, which allows for transport of L. It suggests that more M than L will be absorbed in a healthy gut; therefore, M excretion is used for normalization of L excretion, and an inverse relationship between the absorption of these two sugars is expected [46]. However, in our study, a moderate positive correlation between the excretion of L and M was observed (r = 0.594, *p*-value = 0.001), very close to correlation observed by Ordiz et al. [46] in a rural population of children in Malawi. The authors challenged the interpretation of SAT, and considering M as a normalization parameter, suggesting that, if there is a leak in the intestinal barrier, then both sugars will be absorbed to a higher extent. Doubts in L/M ratio were also addressed in a study on irritable bowel syndrome [11]. The authors highlighted that in a situation of increased excretion of both sugars, the ratio will remain unchanged, suggesting healthy gut; therefore, L/M ratio alone would give false-positive results and fail to detect the leaky gut. In CD, the surface of the villus is reduced, therefore a decrease of M excretion is observed [47], which may affect the L/M ratio. Thus, the increase in the M excretion is the first sign of epithelium recovery in CD after the GFD implementation [47]. Noteworthy is that, besides barrier integrity, several factors can influence the absorption of sugars by epithelial cells, including motility, use of medications and drugs, variation in gastric emptying, intestinal surface area and transit time, and renal clearance [12]. To avoid misinterpretation of SAT, we decided to look separately at individual components of SAT: M and L excretions.

**Figure 2:**
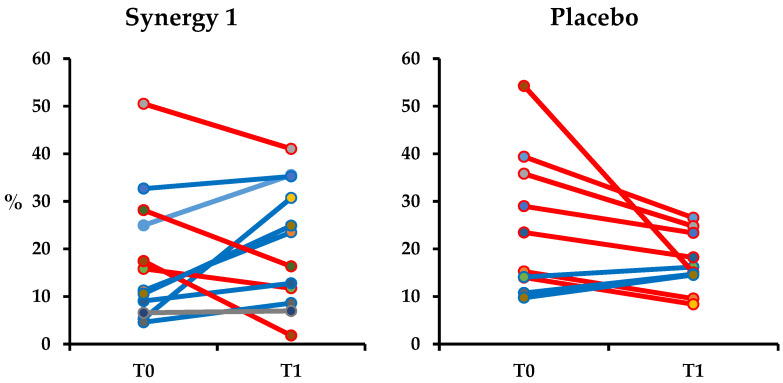
Urinary excretion of mannitol (M) in individuals from Synergy 1 and placebo group, before (T0) and after the intervention (T1), expressed as a percent (%) of total ingested M.

Changes in M excretion in the individual subjects of both experimental groups are presented in Figure 2. At the baseline, values of M excretion for individual subjects varied within the experimental groups; however, there was no significant difference between the mean M excretion between groups (*p*-value = 0.692). In Synergy 1, the baseline mean excretion of M was 18.08 ± 13.86 and in the placebo group, it was 24.59 ± 14.79%. After the intervention, for most of the participants in Synergy 1 group, the M excretion increased resulting in the significant increase in the mean excretion of M (*p*-value = 0.038) in Synergy 1 group, reaching 20.77 ± 12.87% (Figure 2). In the placebo group, a decrease in M recovery was observed for most participants; however, it did not significantly affect the mean M recovery (17.14 ± 6.15%, *p*-value = 0.114). There was a significant difference in the excretion of M between the Synergy 1 and placebo group after the intervention (*p*-value = 0.007). The recovery of M was positively correlated to age (r = 0.450, *p*-value = 0.002) and body mass index (BMI) (r = 0.368, *p*-value = 0.012).

**Figure 3:**
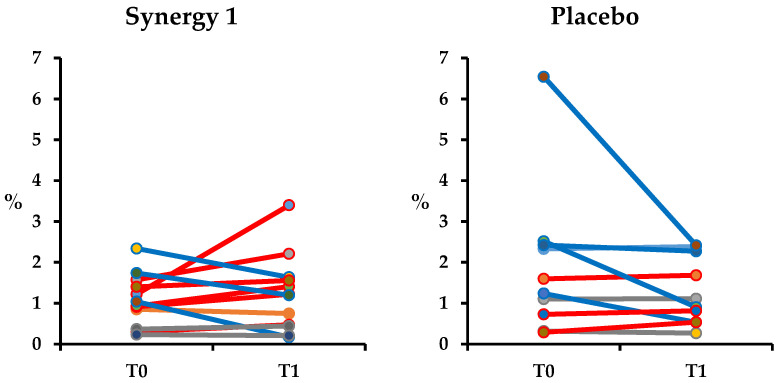
Urinary excretion of lactulose (L) in individuals from Synergy 1 and placebo group, before (T0) and after the intervention (T1), expressed as a percent (%) of total ingested L.

Changes in L excretion in the individual subjects of both experimental groups are presented in Figure 3. At the baseline, the mean values of L excretion in Synergy 1 and placebo group were statistically similar (*p*-value = 0.065) (1.07 ± 0.62 and 1.89 ± 1.82%, respectively). After a twelve-week intake of prebiotic, an increasing tendency in L excretion was observed in the majority of the participants; however, it did not affect the mean value of L recovery (1.22 ± 0.93%, *p*-value = 0.521). In the placebo group a slight, non-significant decrease in the mean L excretion was observed (1.29 ± 0.84%, *p*-value = 0.193). Similarly to M recovery, L was correlated to age (r = 0.269, *p*-value = 0.046) and BMI (r = 489, *p*-value = 0.001). Moreover, L excretion correlated with L/M ratio (r = 0.410, *p*-value = 0.005), which suggests that the L value had a bigger effect on the L/M ratio.

In our study, the opposite phenomena occurred in placebo and Synergy 1 group for sugar recoveries; however, the ratio between L and M remained unchanged in both cases. Therefore, it is important to evaluate the recoveries of individual components of the SAT, not only their ratio. As the amount of M can inform about the extent of an intact epithelial surface area [12], the observed increase in M recovery after the prebiotic intervention may suggest healing of the epithelium. Interestingly, we did not observe any association between L, M, and L/M ratio and glutamine and glutamic acid concentrations presented before [31]. In the previous study, we noticed positive changes in the profile of these amino acids after prebiotic intake. And, as it is known, depletion of intestinal glutamine may result in the atrophy of enterocytes leading to the increased permeability [48]. Even though the positive changes in M was observed, the lack of association cause that the present study cannot confirm the positive tendencies suggested previously [31].

### 3.3. Non-invasive Markers of Intestinal Permeability

The results regarding the non-invasive tests for intestinal permeability evaluation are presented in Table 1. Before the intervention, there were no significant differences in any of the analyzed markers between the experimental groups. After the 12-week nutritional trial, the significant changes were detected in iFABP and zonulin, in both experimental groups. The decrease in iFABP concentration and the increase in zonulin concentration were observed, irrespectively, of the study group, which may confirm positive changes in the intestine during the following of a GFD itself. The supplementation with prebiotic did not influence the other markers of intestinal permeability (Table 1).

iFABP is a sensitive marker of intestinal epithelium damage, being useful in monitoring of enterocyte damage in various diseases, such as necrotizing enterocolitis, intestinal ischemia, and CD [49,50,51]. The damage of enterocytes releases iFABP into circulation; therefore, the level of iFABP informs about the actual extent of epithelial injury [52,53]. In our study, at the baseline, children following the GFD for the shortest time (less than 1 year) had a higher concentration of iFABP (average in both groups: 2.396 ng/mL) in comparison with children staying on a GFD for longer than a year (1.921 ng/mL). There was no further differentiation between GFD treatment for two or more years. Similar findings were reported by Adriaanse et al. [51], who reported that the levels of iFABP decrease during the first months of GFD adherence, reaching the stable concentration after 1–2 years of a GFD regime. Interestingly, in both the cited study and our research, iFABP concentrations do not reach levels observed in healthy controls, even though the tTG values usually normalized within the first two years of treatment [51].

**Figure 4:**
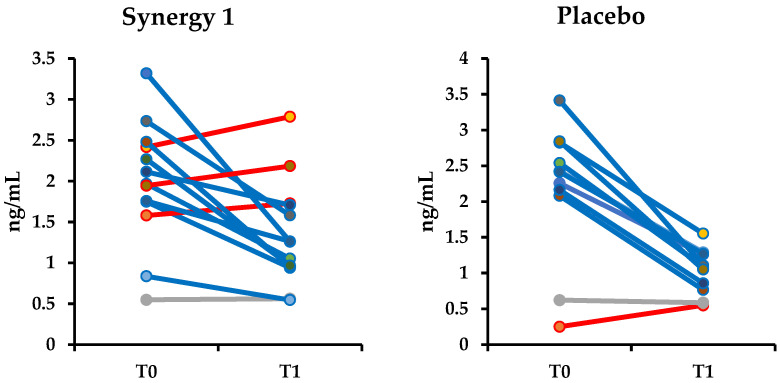
Changes in intestinal fatty acid-binding protein (iFABP) concentrations in the individual subjects from Synergy 1 and placebo group. Increases in the concentration are marked in red.

Irrespectively of the ingested supplement, in both experimental groups, the decrease of iFABP level was observed, suggesting no harmful effect of the applied intervention and further healing of the intestine caused by adherence to GFD. Interestingly, three children demonstrated an increase in the concentration of iFABP after the supplementation with prebiotic (Figure 4). One of these children had GIP present in stool sample after the intervention. It is also possible that the observed increase in the concentration of iFABP was not directly affected by prebiotic itself but may be the result of inadvertent gluten intake. Serum iFABP is used as a biomarker to detect active CD since it is highly expressed in the villi tip. However, iFABP determination may have an advantage over anti-tTG2. Intestinal FABP is a small (14–15 kDa) protein released into the circulation upon enterocyte membrane integrity loss and rapidly excreted into the urine. Thus, iFABP could reflect rapid changes at the mucosal level. Nevertheless, the application of serum iFABP in clinical practice as a marker of epithelium damage requires more studies involving a large number of patients and validated procedures.

Zonulin is a protein released from the lamina propria that alters intestinal permeability; therefore, it is considered as a potential marker of intestinal permeability [54]. In CD, the correlation between zonulin level and intestinal permeability analyzed based on SAT was reported only in patients with Marsh 3 mucosal lesions, while in patients with normal histology this correlation was not observed [55]. Moreover, the authors reported that zonulin may not normalize and remain elevated for many patients with CD who follow GFD, despite the histological normalization [55]. In our study, zonulin increased for all children after the twelve-week intervention, irrespectively, on the supplement intake. Moreover, only a moderate correlation (r = 0.326; *p*-value = 0.040) between zonulin and M recovery was observed, suggesting not so strong association with intestinal integrity. Moreover, zonulin was positively correlated to age (r = 0.056, *p*-value = 0.00004) and BMI (r = 0.581, *p*-value = 00002). Zonulin values noted for children with elevated GIP were not different from values observed for other participants, suggesting that gluten intake did not affect significantly the zonulin level, which was suggested previously [56]. Contrary, Russo et al. [30] reported a decrease in zonulin level after intake of inulin-enriched pasta, which they explained by the selective modulation of intestinal bacteria. However, we are not able to explain why zonulin level increased after the intervention in our study.

A positive moderate correlation was noted also between zonulin and claudin-3 (r = 0.326, *p*-value = 0.031). Claudins are the main components of tight junctions in the epithelium. Claudin-3 is considered as a sealing component of intercellular junctions [57]. Increased expression of claudin-3, similarly to claudin-2, may suggest structural changes of intercellular junctions and may contribute to increased intestinal permeability [58]. Therefore, a decrease in the level of this parameter would be in demand. In our study, no significant effect of the applied supplements on claudin-3 was observed; however, a small decreasing tendency was noted in both experimental groups, as a result of the association to the zonulin.

Another parameter analyzed in our study was GLP-2, which is a gastrointestinal hormone having an intestinotrophic effect [59]. Several animal studies reported the positive effect of GLP-2 on epithelial proliferation and the integrity of the gut barrier [60,61]; therefore, an increase of the GLP-2 level would be desired. In our study, a non-significant but noticeable tendency of increase in GLP-2 was observed in both experimental groups. In the study of Russo et al. [30], intestinal permeability was analyzed in healthy individuals after the ingestion of inulin-enriched pasta, and the increase in GLP-2 concentration was noticed after five weeks of intervention. Similar findings were reported in an in vivo study on obese mice, where the authors noticed that prebiotic added to the mice diet increased circulating GLP-2 and preserved functions of intestinal barrier [61]. The authors explained this phenomenon by selective modulation of gut microbiota, as the prebiotic supplementation increased the number of *Lactobacillus sp*. and *Bifidobacterium sp*. It can explain the lack of differences between the placebo and Synergy 1 groups in our study. As we reported previously [34], the supplementation with Synergy 1 did not affect significantly the intestinal microbiota; therefore, as the association between microbiota modification and GLP-2 level are suggested [61], in our study, the differences were not observed. Noteworthy is that the level of GLP-2 detected by Russo et al. [30] in the study with healthy subjects was almost twice higher (ca. 5 ng/mL) as compared to the values observed in our study. It can be explained by still present micro-injuries in the intestine of children with CD. GFD itself seems not to completely cure the epithelium of CD patients even after many years of adherence, despite the improvement in histological parameters expressed by Marsh scale, which was suggested also in a study evaluating iFABP in adult CD patients [51]. On the other hand, a recent study with children with type 1 diabetes supplemented with oligofructose-enriched inulin reported only small, non-significant increases in GLP-2, similar to our study [43].

We did not observe the effect of prebiotic on calprotectin in stool, a cytosolic protein of neutrophils. When the permeability of the intestinal barrier is increased, granulocytes and monocytes migrate to the gut as the first line of defense in response to the presence of the pathogens which stimulate the release of immunological mediators, including calprotectin secreted by neutrophils [62]. The measurement of calprotectin concentration in the stool is a non-invasive marker of gut inflammation [63] and/or may inform about the intestinal permeability. In our study, calprotectin concentration was moderately correlated to M recovery (r = 0.436, *p*-value = 0.002) and it was the strongest correlation observed between the intestinal state parameters. If we look at individual participants, elevated values of calprotectin informing of inflammation of the intestine (> 100 µg/mg, according to the manufacturer) were noted in four participants from Synergy 1 group before the intervention. After the twelve-week intake of prebiotic, values of calprotectin for three of four children decreased significantly. Interestingly, two of these children were the same for who the increase in iFABP was observed. At the same time, in the placebo group, the opposite phenomenon was observed. In the placebo group, only one child had elevated calprotectin level at the baseline, while, after the intervention, three children had elevated calprotectin level, including two children with calprotectin > 200 µg/mg. Similarly to the effect observed in our study, in patients with ulcerative colitis, who had elevated calprotectin levels, just two-week supplementation with oligofructose-enriched inulin resulted in a significant reduction of calprotectin [64], while the consumption of dietary fiber by healthy individuals with calprotectin levels within a normal range did not affect this parameter [65]. It may explain why we did not observe a difference analyzing whole groups, as most of the children had calprotectin level below 100 µg/mg. It is considered that the level of calprotectin is not elevated in patients with CD [66,67], except for the newly diagnosed children with severe histopathological changes [68]. Therefore, the elevated values may be related to the coexisting inflammation, not CD-associated. The effect observed in three children with elevated calprotectin may suggest a positive effect of prebiotic for individuals with potential ongoing inflammation; however, it requires confirmation in further studies with a bigger number of participants.

In our study, a few limitations should be noted. Firstly, the experimental groups were small and then some of the participants were excluded because of the lack of the required information for the SAT. However, a limited number of participants is related to the preliminary character of our study. We included all the children from the Province Children Hospital, whose caregivers agreed to take part in this trial. We can use this knowledge to calculate the sample size for future, multi-center studies and to better instruct parents to be able to collect complete data. Secondly, we did not analyze other parameters informing of intestinal permeability, i.e., citrulline. However, we think that we analyzed a relatively large number of parameters, related to intestinal permeability in different ways, in a single study, which can answer if the intervention with prebiotics has or has no effect on the intestinal barrier. Another limitation can be a lack of dietary evaluation. We are aware of this point, and we would like to underline that the validated food frequency questionnaires [69] were collected from study participants and will be the subject of a separate manuscript. Despite these limitations, according to our knowledge, our study is the first evaluating the effect of supplementation of GFD with oligofructose-enriched inulin on intestinal permeability in children with CD.

## 4. Conclusions

Our study, for the first time, described the effect of oligofructose-enriched inulin on the intestinal permeability in children with CD treated with a GFD. We found that the supplementation with prebiotic did not have a substantial effect on barrier integrity in this group of patients. Prebiotic intake increased excretion of M in children with CD, which may suggest an increase in the epithelial surface. However, most children seem to have normal values for intestinal permeability tests, confirming the positive changes in the small intestine resulted from the GFD adherence. For individuals with elevated initial values, normalization in L/M ratio and calprotectin was observed after the twelve-week prebiotic intake. However, the number of such participants was too small to make the exact conclusion. Therefore, further studies evaluating the effect of prebiotic on the intestinal permeability in subjects with initial abnormal intestinal permeability, such as newly diagnosed CD patients, are in demand.

## Figures and Tables

**Table 1:** The results of the intestinal permeability markers at baseline (T0) and after the intervention (T1). Quantitative variables with a normal distribution were expressed as mean ± SD, quantitative variables which showed a non-normal distribution were expressed as median (P25–P75).

	T0			T1				
	T0: Placebo Group	T0: Synergy 1 Group	*p*-Value	T1: Placebo Group	T1: Synergy 1 Group	*p*-Value	*p*-Value Placebo T0 vs. T1	*p*-Value Synergy 1 T0 vs. T1
iFABP ^1^ [ng/mL]	2.17 ± 0.94	1.98 ± 0.74	0.59	1.03 ± 0.31	1.35 ± 0.64	0.15	<0.001	0.01
Zonulin [ng/mL]	17.38 (12.82–36.72)	28.66 (2.77–38.64)	0.60	28.82 (18.66–40.77)	36.26 (10.12–44.17)	0.69	<0.01	<0.01
GLP-2 ^2^ [ng/mL]	3.17 (2.44–4.22)	2.23 (1.75-3.67)	0.49	3.99 (3.52–4.51)	3.04 (1.78–3.84)	0.10	0.21	0.81
Claudin-3 [ng/mL]	2.75 ± 0.79	2.41 ± 1.03	0.40	2.44 ± 0.67	2.20 ± 0.63	0.38	0.09	0.46
Calprotectin [µg/mg]	32.95 (18.73–81.72)	30.92 (16.47–136.49)	0.69	44.20 (16.00–108.60)	63.73 (34.24–105.37)	0.64	0.33	0.97

^1^ intestinal Fatty Acid Binding Protein; ^2^ Glucagon-Like Peptide 2.
